# Trichromatic Vat Dyeing of Cationized Cotton

**DOI:** 10.3390/ma14195731

**Published:** 2021-09-30

**Authors:** Ana Sutlović, Martinia Ira Glogar, Ivana Čorak, Anita Tarbuk

**Affiliations:** Department of Textile Chemistry and Ecology, University of Zagreb Faculty of Textile Technology, Prilaz Baruna Filipovića 28a, HR-10000 Zagreb, Croatia; ana.sutlovic@ttf.unizg.hr (A.S.); martinia.glogar@ttf.hr (M.I.G.); ivana.corak@ttf.unizg.hr (I.Č.)

**Keywords:** cationization during mercerization, 3-chloro-2-hydroxypropyltrimethyl ammonium chloride (CHPTAC), vat dyeing, trichromatic dyeing, metamerism, electrokinetic analysis, spectrophotometric analysis

## Abstract

This article deals with cationization of cotton during mercerization and its effects on trichromatic vat dyeing. If cationization is carried out during the after-treatment, regardless of cotton pretreatment, the reaction takes place on the surface and blocks cellulose groups, subsequently resulting in uneven coloration. However, when cationization is carried out with an epihalohydrin during the mercerization process, new cellulose is formed in which the cationic compound is uniformly distributed and trapped between cellulose chains, resulting in uniform coloration after the dyeing process. The reaction time for the process during mercerization is 24 h, thus a more favorable process was researched. Based on electrokinetic analysis, it was found that 5 h was sufficient for the reaction with 3-chloro-2-hydroxypropyltrimethyl ammonium chloride (CHPTAC). The cationization of cotton contributed to the processes of vat dyeing. The change in charge upon cationization resulted in very high adsorption of vat-dye anions, indicating that ionic bonding occurred in addition to van der Waals forces. The color depth improved by more than 10 times. It should be emphasized that the colors with higher chroma and targeted color hue, especially in trichromatic dyeing, were obtained on cationized cotton, in contrast to standard cotton fabrics. The color differences obtained under the different light sources indicate the occurrence of metamerism. Considering the color fastness to laundering, vat-dyed cationized fabrics of all colors may be used in hospitals or other environments where high hygiene and oxidative bleaching are required.

## 1. Introduction

**Figure 1:**
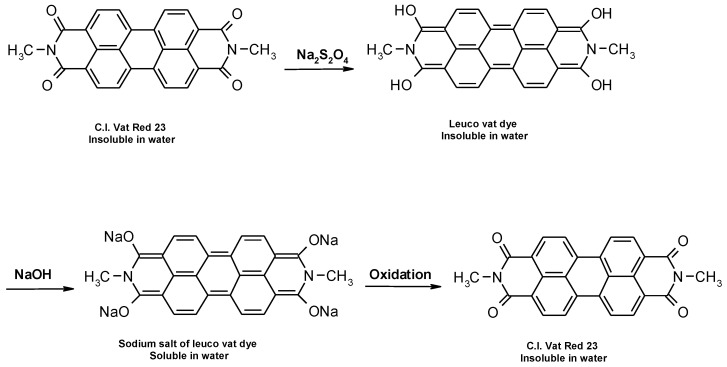
Schematic representation of the reduction and oxidation reaction of vat dye.

Vat dyes are nowadays the most important dye for cellulosic dyeing besides reactive and direct dyes. The dyeing process with vat dyes is highly complex due to their insolubility in water. However, this insolubility is responsible for their satisfactory fastness properties, especially in textile care which requires the use of oxidative bleaches (hospital and other environments with high hygiene requirements). According to their structure, they can be divided into indigoid and anthraquinone dyes, which contain two or more keto groups (C=O), separated by a conjugated system of double bonds. Carbonyl groups affect the substantivity, dye stability, dyeing properties, degree of adsorption, diffusion of the dye into the fiber and color uniformity [1,2,3,4,5]. Due to their insolubility, during the dyeing process they must be converted by reduction from the keto-substituted form to soluble enolate leuco form, in which they are bonding to the textile fiber. The reduction of vat dyes is a reversible reaction, which is illustrated in Figure 1. During oxidation after dyeing, vat dyes are converted back to their insoluble form [1,2,3,4,5,6,7,8,9,10,11].

These dyes are characterized by a change in hue during reduction and oxidation, which is due to the transition of the chromogen from one resonance structure to another, and depends on the degree of oxidation or reduction [1,2,10]. The amount of reducing agent is determined by the chemical structure and nature of a particular dye (number of reducible groups, relative molecular weight, pure dye content) [3,4,5]. This further complicates the dyeing process from the mixture, i.e., the trichromatic dyeing process. Due to the individual molecular structures of the different dyes in the mixture, each dye requires an individual approach in terms of concentration, reducing agent, alkalis, temperature and time. Trichromatic dyeing requires compatibility of the dye components. The requirements for trichromatic dyeing are: dyeing speed, behavior at different electrolyte concentrations, temperatures and pH values, color consistency under different light sources, performance and compatibility with other dyes [12].

The importance of the physicochemical structure of the material in the dyeing processes justifies the significant research in the field of fiber modification and pre-treatment in general. The supramolecular structure (arrangement of crystalline and amorphous regions, microfibrils, nanofibrils) of cotton fiber as a result of growth, level of pre-treatment and modification plays an important role in the vat dyeing process. Scouring and oxidative bleaching of natural cotton results in fiber with cellulose I crystal lattice. The change in the cellulose crystal lattice occurs in the mercerization process and results in cellulose II, which increases the number of available groups in the amorphous region, increasing the dye absorption from 15 to 40% [1,2,13,14,15,16,17]. Cationization, an alternative method to improve adsorption of dyes and anionic auxiliaries, leads to even higher adsorption. Since the cationization process reduces or even eliminates the electrolyte in the dyeing process, many authors refer to it as salt-free or low-salt dyeing [16,17,18,19,20,21,22,23,24,25,26,27,28,29,30,31,32,33,34,35,36,37,38,39].

Research on the cationization of cotton began in 1926 when Kerrer and Wehrli carried out the amination of cellulose. The application of quaternary ammonium compounds began in 1970 with Rupine who treated cotton with a 40% solution of epoxypropyltrimethyl ammonium chloride to achieve better dyeing effects [18], but uniform coloration was not achieved. Since the 1990s, cationic compounds have been investigated to make better exhaustion of anionic dyes (direct and reactive dyes in most cases, acid and metal-complex dyes in some cases, vat dyes in only a few cases) in dyeing and printing [19,20,21,22,23,24,25,26]. Because of its environmental benefits (low-salt dyeing), cationization is intensively researched, as confirmed by nearly 800 published articles and three review articles on cationic agents and techniques in the last decade [29,30,31]. As for as cationic agents are concerned, the application of epihalohydrins, 2,3-epoxypropyltrimethyl ammonium chloride (EPTAC), also known as glycidyltrimethylammonium chloride (GTA), and 3-chloro-2-hydroxypropyltrimethyl ammonium chloride (CHPTAC) still gives the best results with a process time of 24 h. Regarding the techniques of cationization, the pad-batch, exhaustion, pad-steam, and pad-dry-cure (pad-bake) after-treatment techniques were listed in these review articles, but cationization during mercerization was not [29,30,31]. This technique introduced by Croatian researchers Grancarić, Tarbuk and Dekanić in 2003 in slack mercerization of yarn [33,34,35] and further developed in 2009 [36] in mercerization without tension on the jigger resulted in new cotton cellulose, published in 2014 [15,16,37]. When cationization is performed in the after-treatment, it is on the fiber surface and blocks the cellulose groups. When the dyeing process is subsequently carried out, the coloration is not uniform in most cases. However, when cationization with an epihalohydrin is carried out during the mercerization process, new cellulose is formed in which the cationic compound is uniformly distributed and trapped between the cellulose chains, resulting in uniform coloration after the dyeing process [17,36,38]. In the case of cationization during mercerization, direct, reactive, acid and metal-complex dyeing have been researched, whereas vat dyeing has not. In other techniques, vat dyeing was performed after pad-batch or exhaustion cationization, but the coloration was not uniform [10,32].

This is exactly where the research gap was noticed and a systematic study of the application of vat dyes to cotton cationized during mercerization was carried out with the analysis and optimization of all relevant process parameters. Prior to dyeing, the process duration of cationization during mercerization was investigated. It was considered to shorten the time to 5 h instead of 24 h, in order to achieve a more favorable process, but still change the surface charge of the cotton cellulose. Another important aspect in the dyeing processes was also considered, namely trichromatic dyeing. The satisfactory color depth and uniformity achieved with other dyes are certainly the results which confirm the importance of this research, given the existing research gaps in these aspects of vat dye application.

## 2. Materials and Methods

### 2.1. Materials

In this research, a standard cotton fabric (wfk-Testgewebe GmbH, Brüggen, Germany) defined in [39] was used.

Sodium hydroxide p.a. (NaOH), Hydrogen peroxide 30% p.a., Acetic acid 80% p.a. (CH_3_COOH) and Sodium chloride (NaCl) were purchased from Gram-mol d.o.o. (Zagreb, Croatia), Subitol MLF and Lavotan DSU from CHT-Bezema (Montlingen, Switzerland), 3-chloro-2-hydroxypropyl trimethyl-ammonium chloride (CHPTAC) and Sodium dithionite (Na_2_S_2_O_4_) from Sigma-Aldrich (Merck KGaA, Darmstadt, Germany).

Vat dyes from group Indanthren (DyStar Colors Distribution GmbH, Raunheim, Germany) are shown in Table 1.

EMPA ECE reference detergent 77 without optical brightener by Testfabrics, Inc. (West Pittston, PA, USA) was used in washing process for determination of color fastness according to [40].

### 2.2. Treatment Procedure

Standard cotton fabric was cationized during the mercerization process according to Tarbuk et al. [16,17,36]. Different epihalohydrin, a 3-chloro-2-hydroxypropyl trimethyl-ammonium chloride (CHPTAC) was used, and with the reaction time varied: 5 and 24 h. Cationization during mercerization was carried out in a two-step procedure on a jigger (Konrad Peter AG Maschinenfabrik, Liestal, Switzerland) at room temperature: first, mercerization (10 passes in a bath with 24% NaOH, 8 g/L Subitol MLF) was carried out; second, before fixation (hot rinsing), alkaline cotton fabric was cationized in a bath with 50 g/L CHPTAC, sealed and left at room temperature for 5 or 24 h. Such processing on the jigger allows mercerization with 0% tenacity, which means that the fabric was mercerized as slack, but was not allowed to shrink at all. In this case, the fibers were swollen enough to give better luster, higher tensile strength and better adsorption capacity. It was then hot rinsed according to the usual procedure, rinsed and neutralized with 5% acetic acid. For further dyeing, the optimal process of cationization was determined by measuring the electrokinetic potential.

Standard and optimally cationized cotton fabrics were dyed with the Vat dyes (Table 1) by the method of trichromic textile dyeing in a Polycolor (Mathis), finishing and dyeing machine, at 70 °C for 60 min (heating gradient was 3 °C/min). The bath ratio (BR) was 1:30 and the dye concentrations were 1 and 3% owf (over weight of fabric). Based on the diagrams prescribed for the use of vat dyes, the amount of sodium dithionite (Na_2_S_2_O_4_), sodium hydroxide (NaOH) and electrolytes (NaCl) for reduction to leuco form was determined for BR 1:30 for 1% owf dye—Na_2_S_2_O_4_ (1.6 g/L), NaOH (5.25 mL/L), NaCl (11.5 g/L) and for 3% owf dye—Na_2_S_2_O_4_ (2.4 g/L), NaOH (6.75 mL/L), NaCl (19.5 g/L). Before dyeing, the standard cotton fabric was treated with a wetting agent, 1 g/L Lavotan DSU (Bezema), in cold distilled water for 30 min. After dyeing, the fabrics were rinsed, oxidized in hydrogen peroxide, rinsed again, and air dried. As this was the first application of Vat dyes cotton cellulose modified this way, all experiments were carried out in two parallel series; two for standard and two for cationized fabrics.

For the determination of color fastness according to [40], standard and cationized fabrics dyed with 3% owf dye were washed in a Polycolor (Mathis) at 40 °C for 40 min with the program Washtest 40 using 2 g/L EMPA ECE reference detergent 77.

### 2.3. Characterization Methods

The electrokinetic potential (zeta, ζ) was calculated according to the Helmholtz–Smoluchowsky equation after the streaming potential was measured with the SurPASS electrokinetic analyzer (Anton Paar GmbH, Graz, Austria) [41]. The zeta potential (ζ) was measured as a function of pH of the 1 mmol/L KCl using Adjustable Gap Cell, and the IEP (Isoelectric point) was determined. The measurements were performed on two samples. For each point, the streaming potential was measured at 8 points of each fabric for each pH value.

Coloristic analysis was performed by spectrophotometric measurement of colored samples using Datacolor 850 spectrophotometer. All results were measured on samples taken from both series by repeating the measurement procedure at random locations on the samples as suggested in [42]. Thus, the color measurements were made using the Datacolor Tools computer program and “Measuring until tolerance” command, which means that at least 10 measurements must be made, and the results are accepted only if the total color difference between each measurement is less than 0.1 (dE* < 0.1). The obtained results for standard and cationized fabric in relation to the dye concentration are presented as CIEL*a*b* parameters, remission curves and K/S values.

The color depth coefficient K/S was calculated using the following equation:

(1) $$K/S=\frac{{\left(1-R\right)}^{2}}{2R}$$

where R is the remission of the dyed fabric at the wavelength of maximum absorption, K is the absorption coefficient and S is the scattering coefficient.

The total color difference (ΔE_cmc_) between the standard and the tested sample was calculated according to [43] using the following formula:

(2) $$\Delta E_{\mathrm{cmc}}=\sqrt{{\left(\Delta L^{*}/{\mathrm{lS}}_{L}\right)}^{2}+{\left(\Delta C_{\mathrm{ab}}^{*}/{\mathrm{cS}}_{C}\right)}^{2}+{\left(\Delta H_{\mathrm{ab}}^{*}/S_{H}\right)}^{2}}$$

where: ∆E_cmc_ is the total color difference and includes the difference in all color parameters, ∆L* is the difference in lightness, ∆C_ab_* is the difference in chroma, and ∆H_ab_* is the difference in hue. The ellipsoidal semi-axes (lS_L_, cS_c_ and S_H_) provide the ability to interpret the three separate components of color difference (lightness, chroma, and hue) in way that is suitable for a variety of applications. The numerical value of ΔE_cmc_ within tolerance limits (∆E_cmc_ ≤ 2) was used for the evaluation [44].

The analysis of the influence of different light sources was performed for standard light sources “A”—Tungsten lamp, “D65”—Xenon lamp, “F11”—fluorescent light, by calculating the color differences, taking the colors under the standard D65 light as a reference, and comparing them with the colors under the standard lights A and F11.

## 3. Results

**Figure 2:**
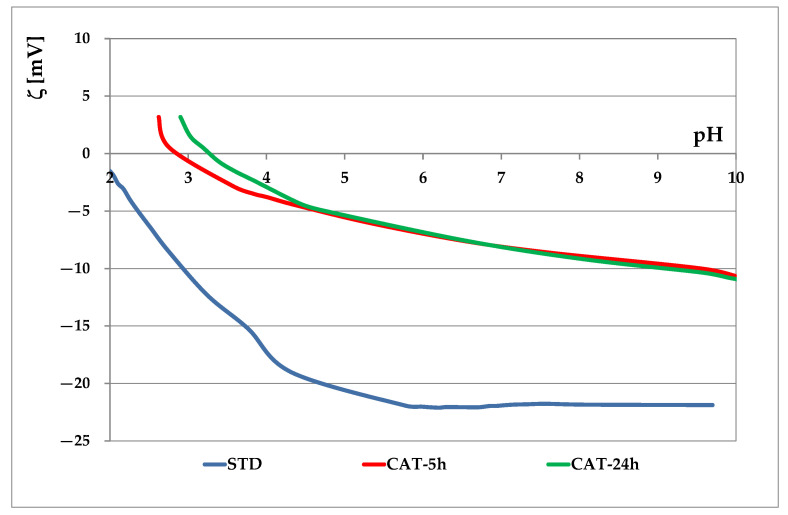
Electrokinetic potential of standard and cationized cotton fabrics vs. pH of 1 mmol/L KCl.

In this work the application of vat dyes to cotton cationized during mercerization was studied. Before dyeing, the process duration of cationization during mercerization was investigated. Thus, cationization during mercerization was carried out with duration of 5 and usual 24 h. In previous studies FT-IR, SEM, TGA, EKA and some other techniques [16] were researched for evaluating the cationization effect. It has been found that in case of cationization during mercerization, mercerization is the dominant process, and the observed changes in FT-IR and SEM contribute to mercerization and are maintained in cationization. However, the change in cellulose was detected by EKA and TGA. Since this work is concerned with the adsorption of leuco-vat anions, the technique of electrokinetic analysis was chosen. The electrokinetic potential of standard and cationized cotton fabrics as a function of pH 1 mmol/L KCl is shown in Figure 2, and the electrokinetic potential at pH 9, pH 6.5 and the isoelectric point are given in Table 2.

From the results in Figure 2 and Table 2 it can be seen that standard cotton fabric is negatively charged due to the presence of hydroxyl and carboxyl groups (ζ = −21.8 mV), as well as carboxyl groups reveled in scouring and bleaching processes [15]. Cationization during the mercerization process leads to a significant modification of fiber surface. Besides –OH and –COOH groups, –NH_2_ groups are also present in cationized fabrics (CAT). Therefore, a higher zeta potential (ζ_CAT-5_ = −9.8 mV, ζ_CAT-24_ = −9.9 mV) was measured, confirming that CHPTAC binds strongly to the surface sites. Comparing the cationization effects after 5 and 24 h, it can be seen that the curves are very similar for both time periods. The only difference can be seen in the acidic medium. However, cotton dyeing occurs in a neutral to alkaline medium where the curves are almost identical. It can be concluded that cationization with CHPTAC does not require a reaction time of 24 h, as suggested in [23], and that it is possible to shorten the process to 5 h.

In the leuco-form, the leuco-vat anion acts similarly to the anion of direct dye, the substantivity of which is mainly responsible for the adsorption of direct dyes. The anions of dissolved reactive dyes, for example, have a lower substantivity [4]. It can therefore be assumed that in vat dyeing, electrokinetics, together with Van der Waals forces, contribute to the adsorption of leuco-vat anions. To confirm this assumption, vat dyeing was performed using the trichromatic method. Trichromatic dyeing is very complex as it is difficult to obtain uniform coloration and satisfactory color depth. Therefore, an analysis of the color depth coefficient (K/S) was performed in parallel with the analysis of the remission characteristics of the obtained colors, since the K/S value is calculated from the color remission.

**Figure 3:**
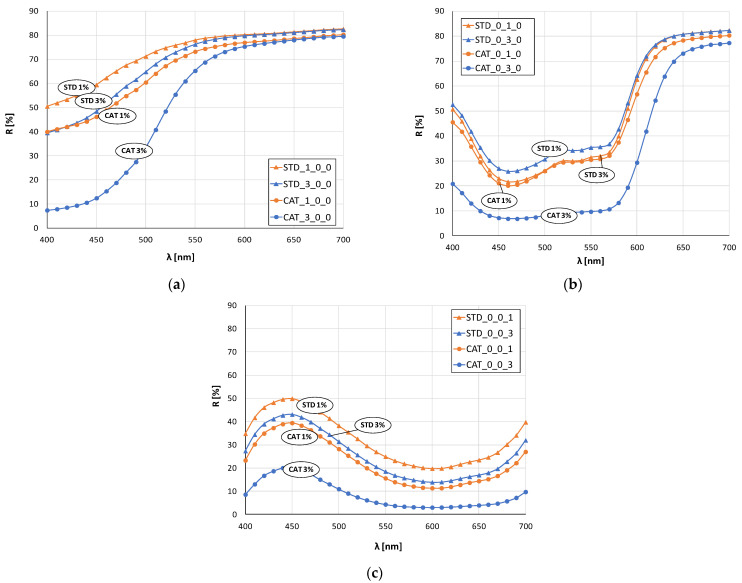
Remission curves of standard and cationized fabrics dyed with 1 and 3% owf dye: (**a**) yellow, (**b**) red, (**c**) blue.

**Figure 4:**
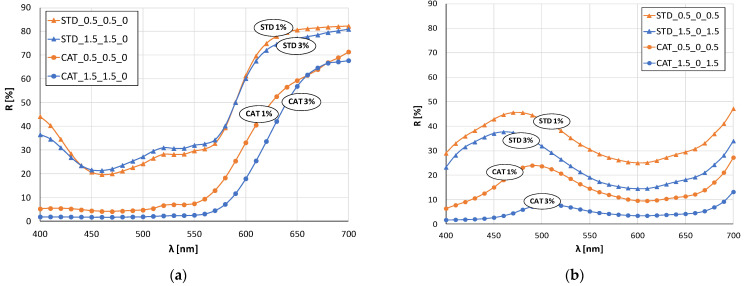

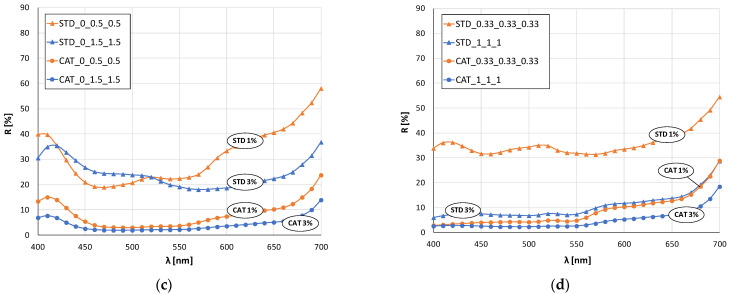
Remission curves of standard and cationized fabrics dyed with 1 and 3% owf mixture of dye: (**a**) yellow and red, (**b**) yellow and blue, (**c**) red and blue, (**d**) yellow, red and blue.

Figure 3 and Figure 4 show the remission values as remission curves, obtained after dyeing with 1 and 3% owf dye. In general, the positive effect of cotton cationization on the affinity of the fibers dye exhaustion is clearly seen in all samples, regardless of whether a dye or dye mixture was applied.

The red color sample (Figure 3b) dyed with 1% owf dye is the only one where there is no significant difference between the standard and cationized fabric. The remissions for 3% owf dye clearly show the positive effect of cationization on dye exhaustion, and low remission values indicate high color depth. For the red color, only the cationized fabric dyed with 3% owf dye shows characteristic peak at a wavelength of about 650 nm, while the other three samples show a bathochromic shift towards the orange spectrum. For the blue color (Figure 3c), the observed differences in heights of the remission curves confirm the difference in color depth between standard and cationized fabrics, for both dye concentrations. A characteristic remission maximum for all fabrics is at 450 nm confirming the uniform color hue, regardless of treatment or dye concentration.

In the case of green color (mixture of blue and yellow dye), there is a clear shift in the maximum of the curves for standard and cationized fabrics (Figure 4b). The maximum of the curve for standard fabrics is still in the blue spectrum (about 480 nm) regardless of the dye concentration, while for cationized fabrics the shift is in the green spectrum (near 500 nm). This confirms the positive effect of cationization on the exhaustion of the yellow dye, which, when mixed with blue, gives the expected green color.

The remission curves for fabrics dyed with a mixture of all three dyes (Figure 4d) show a characteristic color with low intensity and chromatic–achromatic character, except for the standard fabric dyed with a 1% owf dye.

**Figure 5:**
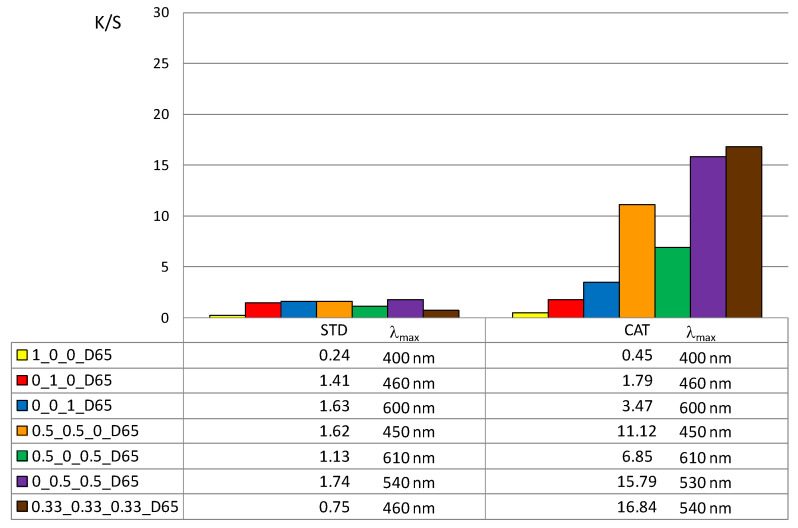
Maximum K/S values of standard and cationized fabric dyed with 1% owf dye.

**Figure 6:**
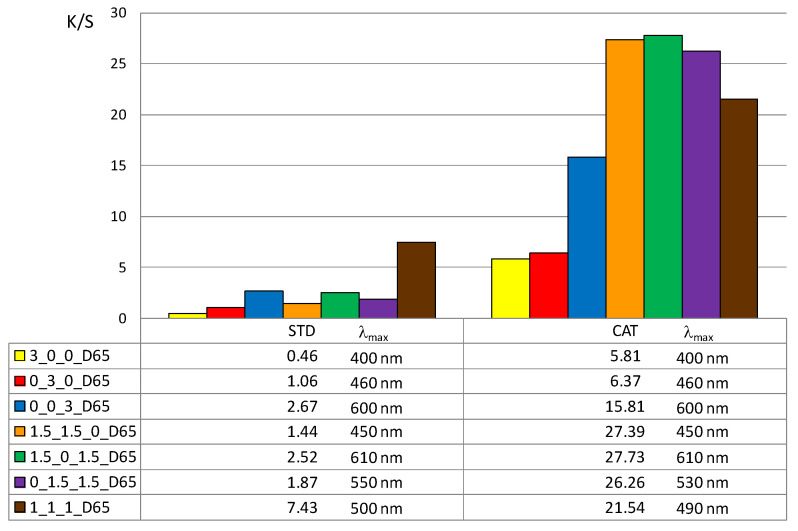
Maximum K/S values of standard and cationized fabric dyed with 3% owf dye.

In addition, the color depth coefficient K/S was calculated, shown in Figure 5 (for 1% owf) and Figure 6 (for 3% owf), together with the corresponding wavelength of maximum absorption.

The K/S values shown in Figure 5 and Figure 6 clearly confirm the positive effect of cotton cationization on dye exhaustion and color depth. The K/S values indicate lower adsorption of vat dye anions on standard than on cationized cotton fabric. When dissolved as leuco-vat dye anions, vat dye follows the general model for sorption and diffusion of the dye anions. The substantivity of vat dye is due to van der Waals forces, which also contribute to the dye anions binding together and forming aggregates, either in solution or after the single dye anion has passed the potential barrier.

Thus, the reason for the low adsorption of standard cotton fabric is the negative charge of the cotton fibers, so that the vat dye anions could not be bound in large amounts. The adsorbed amount of vat dye anions increased with cationization. The change in charge resulted in a very high adsorption of vat dye anions and it was suggested that ionic bonding occurred in addition to van der Waals forces.

The K/S values clearly illustrate the differences in color obtained with 1 or 3% owf of the dye. The color depth is more pronounced with dye mixtures. The K/S values for 1% owf dye are twice as high for one dye and up to 10 times higher for dye mixtures. For 3% owf dye, the K/S values are even higher, 5 times higher for a single dye and up to 20 times higher for dye mixtures. It should be noted that when dyeing cationized cotton, this uptake was the maximum as no visible dye remained in the bath after the dyeing process. Cationization enabled additional ionic bonding which is fully reflected in the exhaustion of dye from the dyeing bath. It is likely that after adsorption in a monolayer, the strong adhesion led to additional layers. The anions are attracted to each other by van der Waals forces and form aggregates. Some of the adsorbed dye was subsequently washed off during soaping, but the much smaller amount of dye goes into the wastewater.

**Figure 7:**
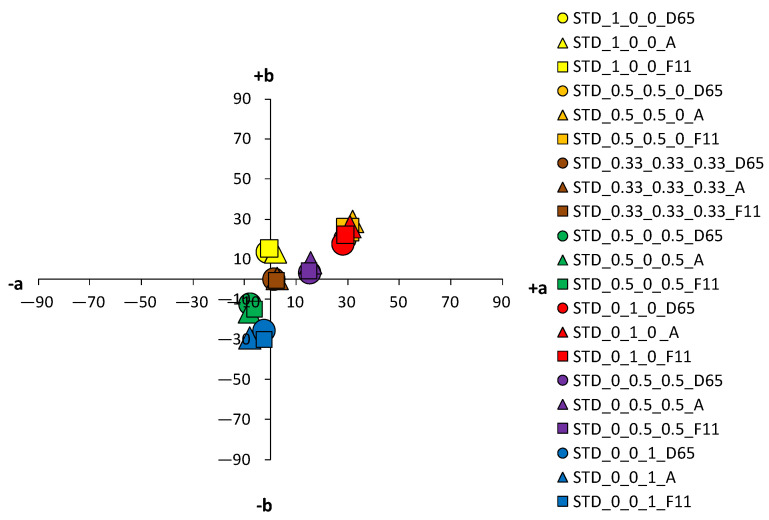
Color coordinates of standard cotton fabric dyed with 1% owf dyes under different light sources.

**Figure 8:**
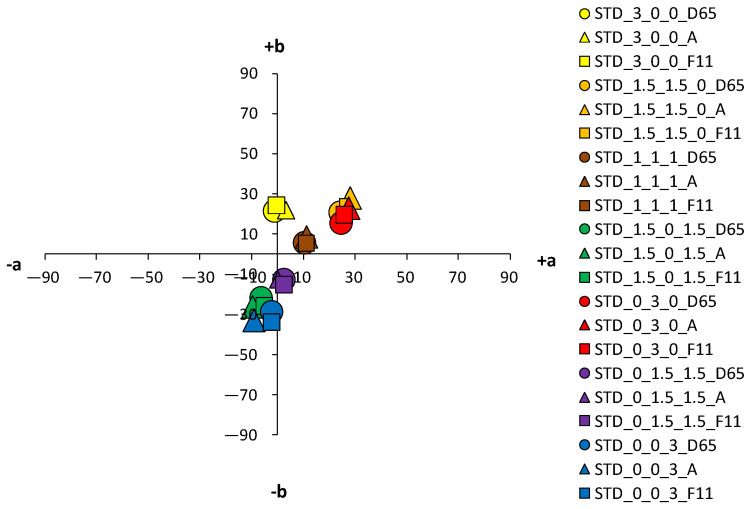
Color coordinates of standard cotton fabric dyed with 3% owf dyes under different light sources.

**Figure 9:**
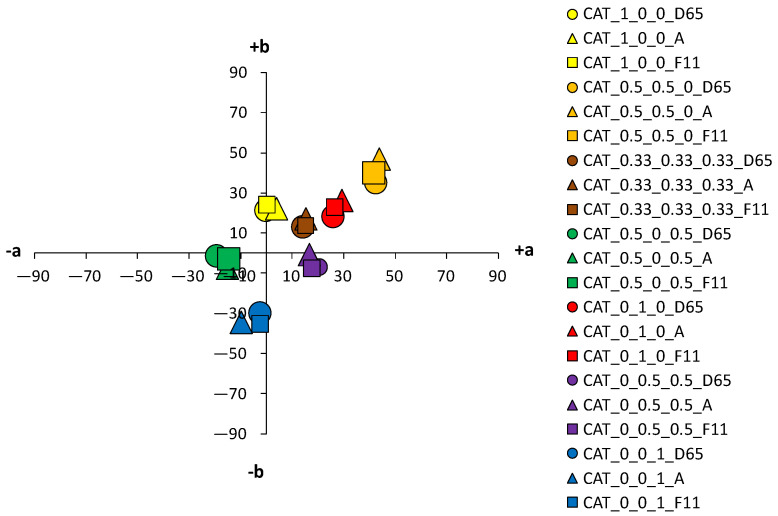
Color coordinates of cationized cotton fabric dyed with 1% owf dyes under different light sources.

**Figure 10:**
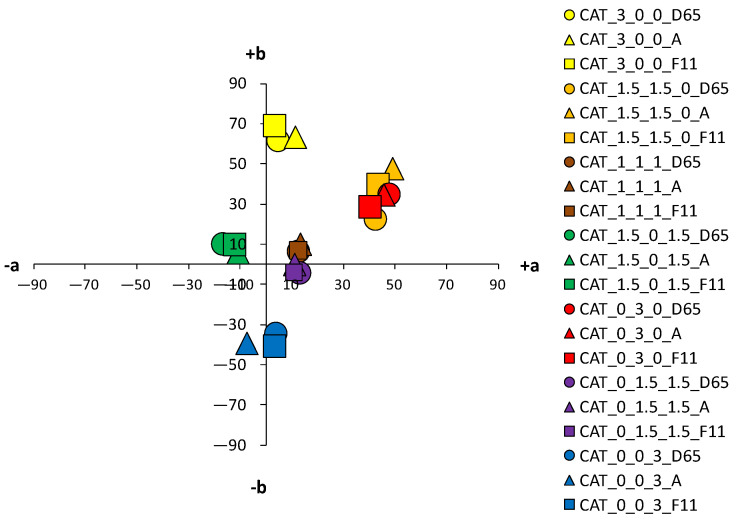
Color coordinates of cationized cotton fabric dyed with 3% owf dyes under different light sources.

An analysis of the coloristic properties of dyed fabrics was also performed. Figure 7, Figure 8, Figure 9 and Figure 10 show the coordinate values of the standard and cationized fabrics, dyed with 1% and 3% owf dye under three different standard light sources: D65, A and F11. By positioning the colors of the samples in the a*/b* color space, the influence of cationization is clearly visible. Higher chroma was obtained for cationized fabric for both concentrations (1 and 3% owf), which is confirmed by the shift of the color from the center of the diagram.

The color parameters of standard and cationized fabrics dyed with 1% owf vat dye are shown in Table 3. For samples dyed in primary color hues (yellow, red, and blue), in the case of yellow coloration, a significant shift in the color hue with respect to cationization is observed from the yellow–green spectrum (standard fabric h = 95.69°) to the yellow spectrum (cationized fabric h = 90.98°). No significant change of color hue with respect to cationization was observed for red and blue coloration.

Fabrics dyed with a dye mixture show a significant effect of cationization on the change in color hue. For the green fabrics dyed with a mixture of yellow and blue dye at concentration of 0.5%, there is a shift in hue from blue-green spectrum of the standard fabric (h = 236.72°) to the green spectrum of the cationized one (h = 183.28°). Analyzing chroma (C*) and hue (h°) of each dye from the mixture, yellow and blue, it is found that cationization significantly affects the exhaustion of the yellow dye compared to blue. Thus, this effect is also seen in the mixture, where the exhaustion of the yellow dye is enhanced in the cationized fabric, giving the expected green color hue. The effect of cationization in a mixture of red and blue dyes is also noteworthy. When the characteristics of chroma (C*) and hue (h°) of red and blue dye were considered individually, no significant changes in the values were obtained with respect to cationization.

However, in the dye mixture, a significant difference in chroma (C*) was obtained for the sample dyed with concentrations of 0.5% for both dyes, which was 15.45 for the standard fabric and 21.51 for the cationized fabric. Additionally, the hue value of a standard fabric dyed with a mixture of red and blue dye is still in the red spectrum (h = 12.79°) and the effect of the blue dye is minimal. Only for the cationized sample, the proportional mixing of the colors is achieved, and the expected red-purple hue is obtained (h = 341.48°), confirming the positive effect of cationization on the exhaustion of the blue dye. For a sample dyed with a mixture of all three dyes (yellow, red and blue) in equal proportions, the achromatic coloration was expected to be in the center of the a*/b* diagram. Due to the apparent weaker exhaustion of the dye, a higher lightness (L* = 64.2), but a rather low chroma (C* = 1.22) was obtained, placing the sample exactly in the center of the a*/b* color space. Such a coloration, regardless of the objective value of the hue in the red spectrum (h = 7.38°), will be visually completely achromatic (gray). As expected, higher adsorption of cationized fabric led to the decrease in lightness (L* = 21.10), but to an increase in chroma (C* = 19.28). This is still a low chroma value, but due to the shift of the color hue into the orange spectrum (h = 43.76°), the sample reaches a brown hue, which in the context of visual perception does not belong to the achromatic but to the achromatic–chromatic range. As for the lightness (L*), the lower values of the cationized samples coincide with the higher chroma values (C*). This is also to be expected, considering that the cationization of cotton increase the affinity of the fibers for dyes and increases the dye exhaustion.

For samples dyed with 3% dye, the differences between the standard and the cationized fabric are even more apparent. In the a*/b* diagram, there is a significant difference in the position of the yellow-colored samples. This is due to a difference in chroma (C*) resulting from a change in color hue with respect to cationization. A significant difference in position was also found for the green samples. The obtained positions of the samples in the a*/b* diagram are consistent and result from the ratio of the objective values of lightness (L*), chroma (C*) and color hue (h°), as shown in Table 4.

As mentioned above, in the group of primaries, the greater differences, indicating a stronger influence of cationization, were found in the yellow-colored samples. However, in contrast to the samples dyed with 1% owf dye, significant differences were observed for both red and blue coloration in the samples dyed with 3% owf dye. The values obtained for lightness (L*) and chroma (C*) follow the theoretical assumptions about the specific ratio of lightness and chroma with respect to the concentration and exhaustion of the dye. As the exhaustion increases, chroma (C*) also increases and lightness (L*) decreases in all samples, with the exception of the green-colored sample.

For the green-colored sample dyed with 3% owf dye, some hue difference occurred on the cationized fabric. The color hue of the standard fabric remained closer to blue and shifted slightly to the green spectrum (h of blue primary = 265.06°; h of green obtained by mixing blue and yellow dye = 253.11°). In the case of the cationized fabric, the significant effect on the adsorption of the yellow dye is confirmed and the color hue is obtained in the green–yellow spectrum (h of blue primary = 276.05°; h of green obtained with a mixture of blue and yellow dye = 148.59°). For the green color, there is also an exception in the chroma value (C*), which decreases for the cationized fabric in contrast to other samples (C* of the standard fabric = 22.47; C* of the cationized fabric = 19.86). This is due to the decrease in lightness (L* of the cationized fabric = 27.5), where the green color, which is naturally medium-light in color, transitions from the chromatic range to the chromatic–achromatic range.

For the purple color hue, there is a difference in coloration behavior between samples dyed in dye concentrations of 1 and 3% owf for the standard fabric. With a mixture of blue and red dye, the obtained color hue shifts only 15.37° towards the blue-violet spectrum (h of primary blue dye = 256.06°; h of the purple obtained with a mixture of blue and red dye = 280.43°). With cationized cotton, the effect of the red dye is more pronounced, and the expected purple color hue is obtained (h = 342.50°). It should be noted that the value of the purple hue obtained for the 3% owf dye corresponds to the hue obtained with 1% owf dye. It is noted that for the standard fabric there is no accentuated difference in the ratio of lightness (L*) and chroma (C*) when the dye concentration is taken into account. However, this difference is more pronounced for cationized fabric. For the sample dyed with a mixture of yellow, red and blue dye at a total concentration of 3% owf, significant differences were observed between the standard fabric and the same fabric dyed with a 1% owf dye. In contrast to the lower concentration, which gave a completely achromatic color, a higher concentration gave a color with more accentuated chroma, close to that of the cationized fabric. The color hue is still in the red–orange spectrum and can be defined in the context of visual experience as chromatic–achromatic brown colors.

An essential part of the color nature is its sensitivity to different spectral distributions of the various light sources. In any more or less accentuated color, there is a change in the basic parameters—lightness (L*), chroma (C*) and hue (h°)—when the light source is changed. This is due to the accentuated differences in spectral content of artificial light sources compared to average daylight. Although metamerism defines the relationship between two samples in terms of a change in light, such changes in individual colors can also be defined as simple metamerism. The effects of different light sources on the standard fabric sample are not significant for both dye concentrations (1 and 3% owf). A slight difference in color parameters with respect to the change of the light source is observed for red and orange samples dyed with 3% owf dye. However, more significant changes in color parameters were obtained with respect to the light source were obtained for cationized fabrics, which were more pronounced for the 3% owf dye.

Since the artificial light source A has an accentuated spectral distribution in the yellow spectrum, there is some yellowing of colors in general, so that the yellow hue moves from the +b* coordinate to the yellow-orange spectrum, the orange hue becomes more yellow, and the blue hue becomes more green, for both dye concentrations. For samples dyed with a mixture of yellow, red and blue dyes (chromatic–achromatic brown hues), there are no significant changes with respect to the light source. For samples dyed with 3% owf dye, a more significant effect of the F11 light source was observed for the yellow, orange and red samples. Namely, the spectral distribution of the F11 light has pronounced peaks in the blue, green and yellow parts of the spectrum. Therefore, in the case of a yellow color hue, it leads to a shift towards the green spectrum, while in the case of an orange color hue, a shift towards the yellow-orange spectrum is observed. In the case of red-orange color hue, a change in chroma is observed and a shift in the coordinate values of the sample for the light source F11 towards the center of the coordinate system (towards the achromatic point) is visible.

**Figure 11:**
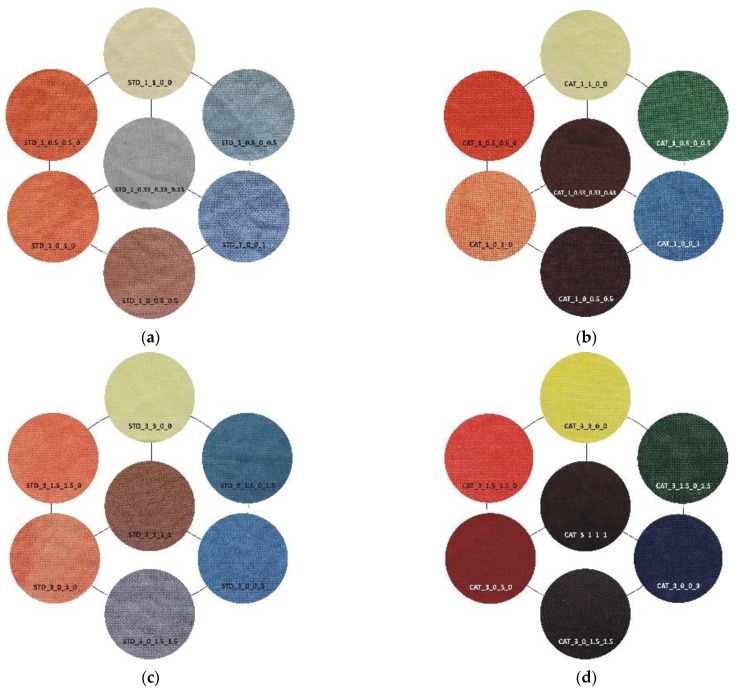
Visual representation of standard and cationized fabric dyed with vat dye: (**a**) Standard fabric—1% owf dye, (**b**) cationized fabric—1% owf dye, (**c**) standard fabric—3% owf dye, (**d**) cationized fabric—3% owf dye.

The visual representation of standard and cationized fabric dyed with 1 and 3% owf dye in Figure 11 clearly confirms this.

To determine the color fastness to domestic and commercial laundering standard and cationized cotton fabric samples dyed with 3% owf vat dye were subjected to one washing cycle at 40 °C with EMPA ECE reference detergent 77 without optical brighteners. Color fastness analysis was performed by calculating the total color difference (∆E_cmc_) between unwashed and washed samples. Metamerism analysis was also performed by calculating the total color differences under different light sources.

From the results presented in Table 5, it can be seen that the values of color differences (∆E_cmc_) are within the tolerance limits (∆E_cmc_ ≤ 2) for most samples, regardless of the dye concentration or fabric treatment. However, the results obtained for standard fabric dyed with a dye mixture (1% owf of each dye) and for cationized cotton dyed with a blue dye and with a mixture of all dyes show that the values of total color difference are slightly above the tolerance limits.

The obtained differences in ∆E_cmc_ values for different light sources indicate the occurrence of metamerism. This is particularly noticeable for a sample dyed with a mixture of yellow, red and blue dye (brown, chromatic–achromatic dye sample). This phenomenon is even more pronounced with cationized cotton fabric. The value of the total color difference of purple-colored fabrics (mixture blue and red dye) is above the tolerance limits for light sources A and F11, but for light source D65 the value of the total color difference is within the tolerance limits.

The color fastness to domestic and commercial laundering is satisfactory. After adsorption and diffusion of the leuco-vat dye anions within the cellulose of fibers, a process of oxidation takes place. Vat dye oxidizes back to the insoluble form and remains trapped in the fiber, so that oxidative bleaching agents added in laundering process do not affect the color. Therefore, such cationized fabrics can be used in hospitals or other environments requiring high hygiene and oxidative bleaching in any desired color.

## 4. Conclusions

Based on the achieved results, it can be concluded:

- Cationization during mercerization can be carried out in 5 h instead of 24 h with similar electrokinetic properties, which is economically advantageous.
- Cotton cationization greatly improved the dye adsorption and contributed to the processes of vat dyeing. In the leuco form, the leuco-vat anion acts similarly to the anion of the direct dye. The substantivity of the vat dye is due to the van der Waals forces, which also help the dye anions to bind together and form aggregates. The charge change during cationization resulted in very high adsorption of the anions of the vat dye, indicating that ionic bonding occurred in addition to the van der Waals forces. The high color depth and the complete adsorption of the dye from the bath confirm this and contribute to the economic and environmentally friendly aspect of this process.
- Although trichromatic dyeing is very complex and some competition may occur between the dyes in the mixture, it has been shown to be possible to perform it with insoluble vat dyes. It is worth highlighting that the colors with higher chroma and targeted color hue, especially in trichromatic dyeing, were obtained on cationized cotton, as opposed to standard cotton fabric.
- The color differences obtained under the different light sources indicate the occurrence of metamerism, which is more pronounced in chromatic cationized cotton. However, under the D65 light, it is within the tolerance limits.
- The color fastness to domestic and commercial laundering is satisfactory. Vat-dyed cationized fabrics in any desired color may be used in hospitals or other environments requiring high hygiene and oxidative bleaching.

## Figures and Tables

**Table 1:** Chemical constitution of used vat dyes.

Dyes	Molecule Chemical Constitution
Indanthren Gelb F3GC Colloisol, C.I. Vat Yellow 12	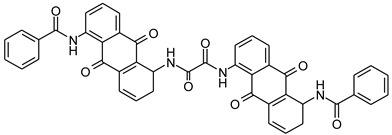
Indanthren Rot FGL Colloisol, C.I. Vat Red 23	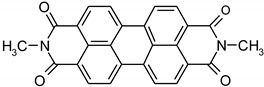
Indanthren Blau CLF Colloisol, C.I. Vat Blue 66	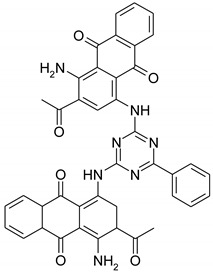

**Table 2:** Electrokinetic potential at pH 9, pH 6.5 and Isoelectric point of standard and cationized cotton fabrics.

Fabric	ζ [mV] at pH 9	ζ [mV] at pH 6.5	IEP	σ	CV [%]
STD	−21.8	−21.7	<2	1.0	6.4
CAT-5 h	−9.8	−7.5	2.88	1.1	4.5
CAT-24 h	−9.9	−7.5	3.26	1.0	4.1

**Table 3:** Color parameters of standard and cationized fabrics dyed with 1% owf vat dye.

	Dyes Concentration(% owf)	L*		C*		h°	
		STD	CAT	STD	CAT	STD	CAT
	1_0_0	90.02	87.23	13.80	21.62	95.69	90.98
	0_1_0	68.55	67.23	33.15	31.64	32.43	35.81
	0_0_1	59.61	49.89	25.61	29.74	264.12	264.81
	0.5_0.5_0	67.55	46.23	35.18	55.11	35.09	39.96
	0.5_0_0.5	63.68	46.09	14.71	19.56	236.72	183.28
	0_0.5_0.5	57.51	26.48	15.45	21.51	12.79	341.48
	0.33_0.33_0.33	64.20	21.10	1.22	19.28	7.83	43.76

**Table 4:** Color parameters of standard and cationized fabrics dyed with 3% owf vat dye.

	Dyes Concentration(% owf)	L*		C*		h°	
		STD	CAT	STD	CAT	STD	CAT
	3_0_0	88.74	81.36	21.82	62.07	93.55	85.88
	0_3_0	70.89	47.80	29.02	47.94	32.84	28.32
	0_0_3	53.22	29.65	28.57	34.45	265.06	276.05
	1.5_1.5_0	68.47	34.87	31.94	59.03	41.28	36.42
	1.5_0_1.5	53.58	27.50	22.47	19.86	253.11	148.59
	0_1.5_1.5	52.45	18.29	12.64	13.50	280.43	342.50
	1_1_1	35.77	21.64	11.84	14.05	30.02	28.15

**Table 5:** Total color difference (∆E_CMC_) between washed and unwashed standard (STD) and cationized (CAT) fabric dyed with 3% owf dye at different light sources.

	Dyes Concentration(% owf)	STD			CAT		
		∆E_cmc_			∆E_cmc_		
		D65	A	F11	D65	A	F11
	3_0_0	1.65	1.62	1.40	0.76	0.69	0.78
	0_3_0	0.32	0.27	0.24	0.72	0.67	0.92
	0_0_3	1.43	1.30	1.38	3.04	2.59	2.98
	1.5_1.5_0	0.75	0.64	0.63	1.44	1.34	1.95
	1.5_0_1.5	2.18	1.91	2.10	0.76	1.11	0.95
	0_1.5_1.5	2.10	2.39	2.23	1.64	2.37	2.19
	1_1_1	3.02	1.78	3.24	3.95	2.58	4.56

## Data Availability

Data available in a publicly accessible repository.

## References

[1] Latham F.R., Shore J. (1995). Dyeing with vat dyes. Cellulosics Dyeing, Chapter 5.

[2] Parac-Osterman Đ., Karaman B. (2013). Osnove Teorije Bojenja Tekstila (Eng. Theory of Textile Dyeing).

[3] Božič M., Kokol V. (2008). Ecological alternatives to the reduction and oxidation processes in dyeing with vat and sulphur dyes. Dye Pigment.

[4] Aspland J.R. (1997). Textile Dyeing and Coloration.

[5] Baumgarte U. (1987). Reduction and oxidation processes in dying with vat dyes. Melliand Text..

[6] Patra S.K., Patra A.K., Ojha P., Shekhawat N.S., Khandual A. (2018). Vat dyeing at room temperature. Cellulose.

[7] Teli M.D., Paul R., Landage S.M., Aich A. (2001). Ecofriendly processing of sulphur and vat dyes—An overview. Indian J. Fibre Text. Res..

[8] Roessler A., Jin X. (2003). State of the art technologies and new electrochemical methods for the reduction of vat dyes. Dye. Pigment..

[9] Lee J.J., Shim W.S., Kim I.S., Kim J.P. (2005). Dyeing and Fastness Properties of Vat Dyes on a Novel Regenerated Cellulosic Fiber. Fibers Polym..

[10] Moustafa A.B.E., Kh E., Saleh S.M. (2011). Improving Dyeability of Cotton Fabric for Vat Dyes. Res. J. Text. Appar..

[11] Santhi P., Jeyakodi M.J. (2010). Study on different reducing agents for effective vat dyeing on cotton fabric. Indian J. Fibre Text. Res..

[12] Mijač J. (2017). Trichromic in the Process of Dyeing Polyamide Knitting with Acid Dyes. Bachelor’s Thesis.

[13] Soljačić I., Žerdik M. (1968). Cotton mercerization. Tekstil.

[14] Dinand E., Vignon M., Chanzy H., Heux L. (2002). Mercerization of Primary Wall Cellulose and its Implication for the Conversion of Cellulose I to Cellulose II. Cellulose.

[15] Stana-Kleinschek K., Strand S., Ribitsch V. (1999). Surface Characterization and Adsorption Abilities of Cellulose Fibers. Polym. Eng. Sci..

[16] Tarbuk A., Grancarić A.M., Leskovac M. (2014). Novel cotton cellulose by cationisation during the mercerisation process—Part 1: Chemical and morphological changes. Cellulose.

[17] Tarbuk A., Grancarić A.M., Leskovac M. (2014). Novel cotton cellulose by cationisation during mercerisation—Part 2: Interface phenomena. Cellulose.

[18] Rupin M., Veatue J., Balland B. (1970). Utilization of reactive epoxy-ammonium quaternaries on cellulose treatment for dyeing with direct and reactive dyes. Textilveredlung.

[19] Lewis D.M., McIlroy K.A. (1997). The Chemical Modification of Cellulosic fibers to Enhance Dyeability. Rev. Prog. Color.

[20] Hauser P.J., Tabba A.H. (2001). Improving the Environmental and Economic Aspects of Dyeing Cotton. Color. Technol..

[21] Cannon K.M., Hauser P.J. (2003). Color Assessment of Cationic Cotton Dyed with Fiber Reactive Dyes. AATCC Rev..

[22] Hashem M., Hauser P., Smith B. (2003). Reaction Efficiency for Cellulose cationization using 3-Chloro-2-Hydroxypropyl Trimethyl Ammonium Chloride. Text. Res. J..

[23] Kanik M., Hauser P.J. (2002). Printing of Cationized Cotton with Reactive Dye. Color. Technol..

[24] Draper S.L., Beck K.R., Smith C.B., Hauser P. (2003). Characterization of the Dyeing Behavior of Cationic Cotton with Acid Dyes. AATCC Rev..

[25] Draper S.L., Beck K.R., Smith C.B. (2002). Characterization of the Dyeing Behavior of Cationic Cotton with Direct Dyes. AATCC Rev..

[26] Hashem M. (2006). Development of a One-stage Process for Pre-treatment and Cationization of Cotton Fabric. Color. Technol..

[27] Ma W., Shen K., Xiang N., Zhang S. (2017). Combinative Scouring, Bleaching, and Cationization Pretreatment of Greige Knitted Cotton Fabrics for Facilely Achieving Salt-Free Reactive Dyeing. Molecules.

[28] Acharya S., Abidi N., Rajbhandari R., Meulewaeter F. (2014). Chemical cationization of cotton fabric for improved dye uptake. Cellulose.

[29] Aktek T., Millat A.K.M.M. (2017). Salt free dyeing of cotton fiber—A critical review. Int. J. Text. Sci.

[30] Correia J., Rainert K.T., Oliveira F.R., Valle R.C.S.C., Valle J.A.B. (2020). Cationization of cotton fiber: An integrated view of cationic agents, processes variables, properties, market and future prospects. Cellulose.

[31] Choudhury A.K.R. (2014). Coloration of Cationized Cellulosic Fibers—A Review. AATCC J. Res..

[32] Fu S. (2016). Studies on Dyeing Cationized Cotton. Ph.D. Thesis.

[33] Grancarić A.M., Tarbuk A., Dekanić T. (2004). Elektropozitivan pamuk (eng. Electropositive Cotton). Tekstil.

[34] Lisec T. Electrokinetic Potential of Cationized Cotton. Master’s thesis, University of Zagreb Faculty of Textile Technology, Zagreb, Croatia, 2003.

[35] Grancarić A.M., Tarbuk A., Jančijev I., Caivano J.L., Lopez M. (2006). Dyeing Effects of Cationized Cotton. Color: Ciencia, Artes, Proyecto y Enseñanza.

[36] Tarbuk A. Interface Phenomena of Cationized Cotton. Ph.D. thesis, University of Zagreb Faculty of Textile Technology, Zagreb, Croatia, 2009.

[37] Tarbuk A., Grancarić A.M., Mondal I.H. (2015). Interface Phenomena of Cotton Cationized in Mercerization. Cellulose and Cellulose Derivatives: Synthesis, Modification and Applications, Part I: Cellulose Synthesis and Modification, Chapter 6.

[38] Tarbuk A., Sutlović A., Dekanić T., Grancarić A.M., Zdjelarević I., Simončič B., Tomšič B., Gorjanc M. (2016). Ultra-Deep Black Cationized Cotton by Metal-Complex Dyeing. 16th World Textile Conference AUTEX 2016—Proceedings.

[39] DIN 53919-1:1980 (1980). Test Cotton Fabrics for Laundering Process Control, Requirements.

[40] ISO 105-C06:2010 (2010). Textiles—Tests for Colour Fastness—Part C06: Colour Fastness to Domestic and Commercial Laundering.

[41] Grancarić A.M., Tarbuk A., Pušić T. (2005). Electrokinetic Properties of Textile Fabrics. Color. Technol..

[42] ISO 105-J01:1997 (1997). Textiles—Tests for Colour Fastness—Part J01: General Principles for Measurement of Surface Colour.

[43] ISO 105-J03:2009 (2009). Textiles—Tests for Colour Fastness—Part J03: Calculation of Colour Differences.

[44] Parac-Osterman Đ. (2007). Osnove o Boji i Sustavi Vrjednovanja, (Eng. Color Basics and Evaluation Systems).

